# Effect of socioeconomic factors during the early COVID-19 pandemic: a spatial analysis

**DOI:** 10.1186/s12889-022-13618-7

**Published:** 2022-06-18

**Authors:** Ian W. Tang, Verónica M. Vieira, Eric Shearer

**Affiliations:** 1 Department of Environmental and Occupational Health, Program in Public Health, Susan and Henry Samueli College of Health Sciences, University of California, Irvine, 100 Theory Drive, Irvine, CA 92617 USA; 2 Communicable Disease Control Division, Orange County Health Care Agency, Santa Ana, USA

**Keywords:** social determinants, COVID-19, spatial analysis, census tracts

## Abstract

**Background:**

Spatial variability of COVID-19 cases may suggest geographic disparities of social determinants of health. Spatial analyses of population-level data may provide insight on factors that may contribute to COVID-19 transmission, hospitalization, and death.

**Methods:**

Generalized additive models were used to map COVID-19 risk from March 2020 to February 2021 in Orange County (OC), California. We geocoded and analyzed 221,843 cases to OC census tracts within a Poisson framework while smoothing over census tract centroids. Location was randomly permuted 1000 times to test for randomness. We also separated the analyses temporally to observe if risk changed over time. COVID-19 cases, hospitalizations, and deaths were mapped across OC while adjusting for population-level demographic data in crude and adjusted models.

**Results:**

Risk for COVID-19 cases, hospitalizations, and deaths were statistically significant in northern OC. Adjustment for demographic data substantially decreased spatial risk, but areas remained statistically significant. Inclusion of location within our models considerably decreased the magnitude of risk compared to univariate models. However, percent minority (adjusted RR: 1.06, 95%CI: 1.06, 1.07), average household size (aRR: 1.06, 95%CI: 1.05, 1.07), and percent service industry (aRR: 1.05, 95%CI: 1.04, 1.06) remained significantly associated with COVID-19 risk in adjusted spatial models. In addition, areas of risk did not change between surges and risk ratios were similar for hospitalizations and deaths.

**Conclusion:**

Significant risk factors and areas of increased risk were identified in OC in our adjusted models and suggests that social and environmental factors contribute to the spread of COVID-19 within communities. Areas in north OC remained significant despite adjustment, but risk substantially decreased. Additional investigation of risk factors may provide insight on how to protect vulnerable populations in future infectious disease outbreaks.

## Introduction

Since the first local case of novel coronavirus 2019 (COVID-19) was identified in January 2020, Orange County (OC), California, has experienced several distinct surges of disease transmission. The first wave occurred in June and July of 2020 (surge 1), followed by a second and more intense wave spanning November 2020 to January 2021 (surge 2). During both surges, populous cities of Santa Ana and Anaheim recorded higher incidence rates compared to the rest of the county. The local health department, Orange County Health Care Agency (OCHCA), reported that Anaheim accounted for 17% of all cases during both surges, and Santa Ana accounted for 19% and 18% during surge 1 and 2 respectively [1]. Furthermore, COVID-19 has reemphasized the already existing disparities and health inequities amongst minority populations. During surge 1, Hispanic cases made up a higher proportion (64%) of community deaths compared to Non-Hispanic White (21%) and Non-Hispanic Asian (11%). A similar trend was observed during surge 2, where Hispanic cases made up 46% of community deaths compared to Non-Hispanic White (29%) and Non-Hispanic Asian (22%) [1]. These health disparities were noted by county health officials and were observed to differ spatially; therefore, social determinants of disease may explain spatial variability of COVID-19.

The relationship between social determinants of health and communicable diseases has been documented prior to the COVID-19 pandemic. During a 2009 outbreak of measles in Romania, public health epidemiologists concluded that “minority groups and living in close communities were at higher risk of measles infection” [2]. A European study found an inverse relationship between public wealth and prevalence rates of tuberculosis amongst EU member states [3]. Additionally, the clustering of COVID-19 cases in OC suggest considerable variability within a county and prior studies in OC have observed disparities in low-income, disadvantaged, and minority communities [4–6]. These disparities may be compounded in the community due to factors including inadequate health insurance coverage, limited access to care, poor health literacy, employment in high-exposure jobs, and housing insecurity and overcrowding, suggesting that social factors exacerbate pandemics [7–14]. Many observational studies are unable to account for confounding by occupation, education, and housing status which may increase the likelihood of COVID-19 outcomes [11]. These factors were influenced by the Commission on Social Determinants of Health (CSDH) framework by the World Health Organization in which socioeconomic positions are interplaying with societal policies and governance. Therefore, structural inequalities are to be addressed in order to limit communicable diseases that are exacerbated by social factors [15]. The spread of an infectious disease is spatial in nature, and clustering of COVID-19 cases has been documented around the world [16]. Many of these studies use large spatial scales, such as provinces, or counties, but social and environmental determinants of health may vary widely within these large regions [13, 17–21].

Initial surveillance from OCHCA observed more cases in specific communities in the OC, potentially suggesting spatial clustering of COVID-19 outcomes. Therefore, using readily available patient data captured in public health surveillance systems, this study seeks to investigate the spatial distribution of COVID-19 cases in the context of social determinants of health associated with COVID-19 transmission, hospitalization, and death using census-tract data. We used generalized additive models to analyze COVID-19 census-tract level spatial patterns in an ecological study and assess whether community risk factors such as race/ethnicity, service occupation, household size, and age contributed to those patterns. This study benefits from using data from the start of COVID-19 pandemic and does not include variants, rapid-testing, and only incorporated early vaccine distribution. It also aims to investigate COVID-19 at a finer spatial resolution than previous studies and how specific community factors impact COVID-19 outcomes.

## Methods

### Study population and area

Confirmed COVID-19 cases are reported to OCHCA through the state-wide public health reporting system for reportable diseases. Confirmed cases were cases that had a positive polymerase chain reaction (PCR) result. This study included cases which had specimen collected dates from January 28^th^, 2020 to February 28^th^, 2021. Data included the residential location of each case, whether they were hospitalized, and whether they died from COVID-19. Additional race/ethnicity data for cases were not considered for this analysis due to missingness. Cases missing longitude and latitude (6.6%) and specimen collected date (< 0.01%) were not included in this analysis. We also excluded cases outside of OC, that were unable to be geocoded, incarcerated, long-term care facility residents, and homeless shelter residents (4.0% total) in order to identify risk within the community. Therefore, 221,843 cases were analyzed in order to observe cases in the community. This study examined the effect of location using census tracts within OC. Census tracts are small subdivisions within the county that have between 1,200 to 8,000 people, with an optimum size of 4,000 people, making it an ideal unit of measure for a local municipality. The size is often determined by the area of the census tract relative to the population, and therefore smaller area census tracts represent higher population density, and larger area census tracts have lower density [22]. The number of COVID-19 cases were aggregated for the 583 OC census tracts, and the X and Y coordinates of each census tract were then used in our analysis to estimate the log risk across location. This study was reviewed and approved by the OCHCA IRB Review Board.

### Community variables

We assessed the effect of community risk factors on location using data collected by the 2019 Census American Community Survey (ACS) and included variables which we hypothesized contributed to COVID-19 risk at the census-tract level. COVID-19 has disproportionately affected some populations more than others [23–25]. We calculated the percent of minority at each census tract, defined as the sum of Black, Hispanic/Latino, Asian, Mixed-Race, and Other. Percent of individuals in the service industries were included to capture those who were in high-risk occupations and may not be able to socially-distance during state-wide stay-at-home orders [12, 26]. Average household size was also included to account for household spread, and median household income was used as a surrogate for education and as an indicator for the ability to stay at home [27–29]. Lastly, we also included percent 65 years and older at each census tract to also capture high-risk groups [30, 31]. We examined percent with a high school education and above as a risk factor of interest in our univariate analyses but excluded it from our full analysis due to multicollinearity issues.

### Statistical analysis

We investigated the relationship between COVID-19 and location in two analyses: a primary analysis with COVID-19 cases, and a secondary analysis with hospitalization and deaths from COVID-19. We separated our primary analysis into three time periods: the full study period (March 1, 2020 to February 28, 2021), the first half of the study period which contained surge 1 (March 1, 2020 to August 31, 2020) and second half of study period which contained surge 2 (September 1, 2020 to February 28, 2021). Only one case occurred between January 28, 2020 and March 2020, and we therefore included that case in the full analysis and the analysis of the first half of the pandemic. Each time period captured a peak surge and a period of case depression.

We used generalized additive models (GAM) within a Poisson framework to analyze the spatial association between location and confirmed COVID-19 cases while simultaneously adjusting for community variables using the MapGAM package (version 1.2–5) in R (version 4.0.2). Location in this model is the centroid of a census tract and accounts for each census tract’s population relative to its area using an offset in the Poisson framework. GAMs estimate the log risk of COVID-19 at a specific location by applying a locally weighted straight line smoother (LOESS) to smooth over location (longitude and latitude). The degree of smoothing, or optimal span size, is determined by minimizing the Akaike’s Information Criterion (AIC). The span size refers to the percent of data that is being weighted as a distance of function. For example, a span size of 0.20 would use 20% of the nearest data from a point on the map to calculate the log disease risk. The smaller the span size, the more variation of risk is displayed while larger span sizes create a smoother surface. Without the smoothing term, the GAM is a regular GLM model. The GAM framework is defined as:

$$\mathrm{Log}\left[\mathrm{p}\left(\mathrm{X},\mathrm{Y}\right)\right]=\mathrm{S}\left(\mathrm{X},\mathrm{Y}\right)+\mathrm{offset}\left(\mathrm{pop}\right)+\mathrm{\alpha };\mathrm{ \alpha }=\upbeta 0+{\upbeta }_{1}{\mathrm{Z}}_{1}+{\upbeta }_{2}{\mathrm{Z}}_{2}+\dots +{\upbeta }_{\mathrm{j}}{\mathrm{Z}}_{\mathrm{j}}$$

where Log[p(X,Y)] is the log disease risk at a census tract centroid, S(X,Y) is the smoothing function of location at centroid position X,Y, offset(pop) is the population size of the census tract, and α are covariate Z with their respective β coefficients. The GAM model will predict the log risk for all possible combinations of X and Y centroids in the study area. The centroids of census tracts are then randomly permuted 1000 times across the study area to test whether location is significant globally and locally. The global test of location is calculated by comparing the deviance of a model with and without the smoothing term, while local significant values rank the lower and upper 2.5% of the permuted pointwise distribution. Risk is therefore indicated as the z-dimension, denoted by color on the map, across a two-dimensional surface representing X and Y.

For our GAM maps, we report crude models which includes only the smoothing function for location and population size, and the adjusted model which includes the smoothing term for location, population size, and our covariates. The adjusted models also produce risk ratios for covariates, providing an effect estimate for the association between that variable and COVID-19 outcomes. A “flat” map with no variation in color signals that our covariates explained some of the spatial variability of COVID-19 cases. In addition, we report effect estimates from standard generalized linear models (GLM) with a Poisson distribution used in our univariate analyses for each of the covariates (i.e., without the smoothing function) to observe risk ratios without accounting for spatial location. In total, we produce maps illustrating spatial risk in a crude GAM model and an adjusted GAM model; we also report effect estimates for model covariates with a univariate GLM model and an adjusted GAM model that includes a smoothing term for location (same model that produces the map).

## Results

**Fig. 1:**
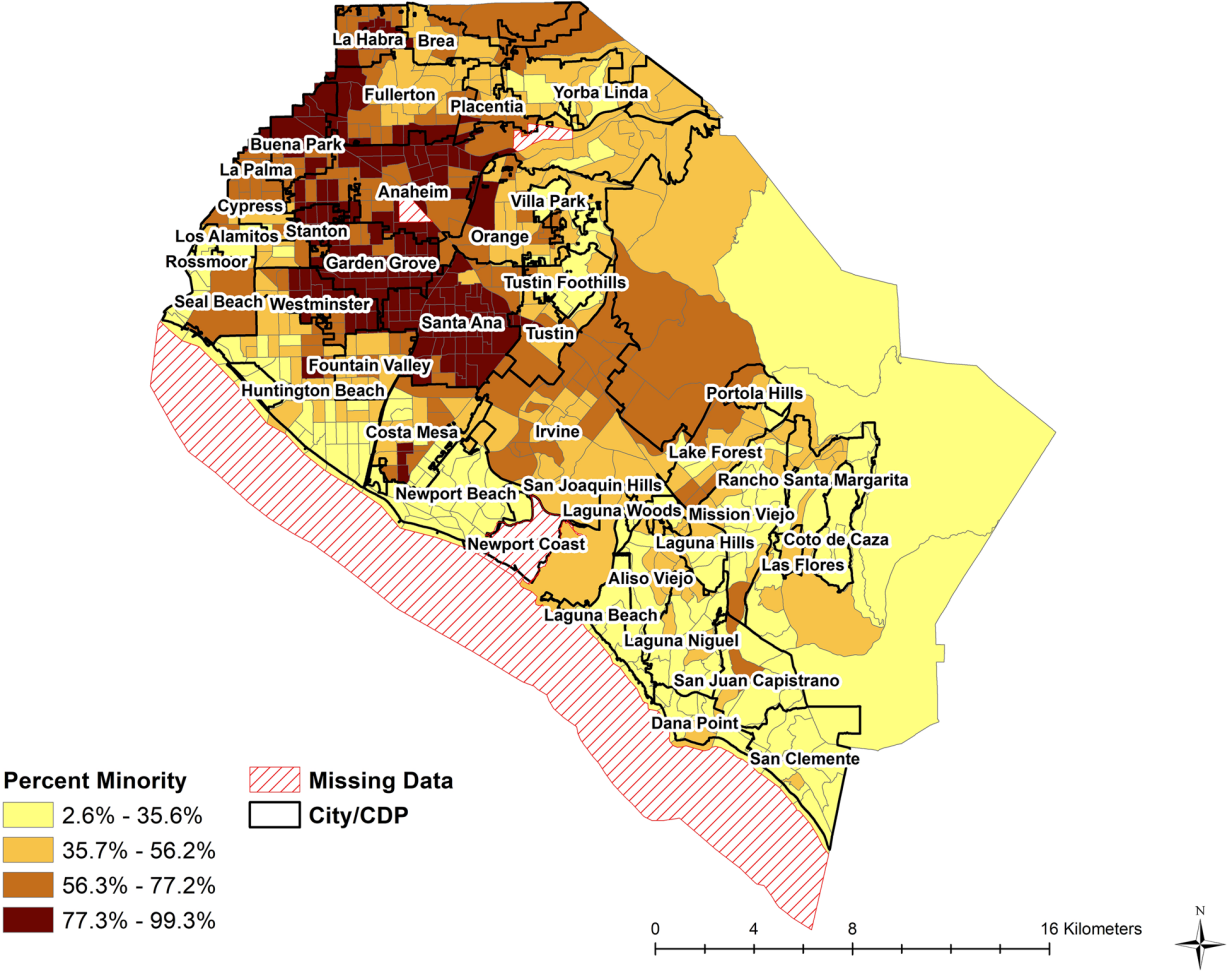
Cities and distribution of minority populations by Census Tracts, Orange County, CA, 2019. CDP: Census Designated Place

A total of 221,843 cases were included in our analysis across our entire study period. These cases were geocoded and analyzed to 579 of 583 census tracts within OC; there were 4 census tracts that were missing either race/ethnicity data, household income, average household size, and education data (Fig. 1). In these missing census tracts, there were 266 cases, 14 hospitalizations, and 4 deaths were thereby excluded from the analysis. These census tracts included the ocean, Disneyland, Newport Coast, and a recycling center. At the start of the pandemic (surge 1), cases ranged from 2–369 cases per census tract, and increased to 9 to 1,230 cases by the second half (surge 2) of the study period (Table 1). Across the entire study period, hospitalizations ranged from 0 to 68, and averaged at 14.5 per census tract while deaths ranged from 0 to 26 and averaged at 4.7 per census tract. Demographic data among all census tracts had wide ranges, illustrating the diversity of communities in OC (Table 1, Fig. 1).

**Table 1:** Characteristics of Orange County Census Tracts, March 2020- February 2021

Characteristic	Entire County, Mean ± SD	Range
Cases	382.6 ± 257.5	11–1519
Cases- Surge 1^a^	53.0 ± 57.8	2–369
Cases- Surge 2^b^	310.0 ± 203.0	9–1230
Hospitalizations	14.5 ± 11.2	0–68
Deaths	4.7 ± 4.4	0–26
Percent Minority	56.3 ± 24.1	2.6–99.3
Average Household Size	3.1 ± 0.7	1.4–5.8
Percent Service Industry	17.0 ± 7.6	0.7–43.0
Median Household Income	95,812 ± 34,228	26,750–220,286
Percent Population 65 +	15.7 ± 9.6	2.0–83.7
Percent HS degree +	85.7 ± 13.7	33.5–100

^a^March 1, 2020- August 31, 2020

^b^September 1, 2020-February 28, 2021

**Fig. 2:**
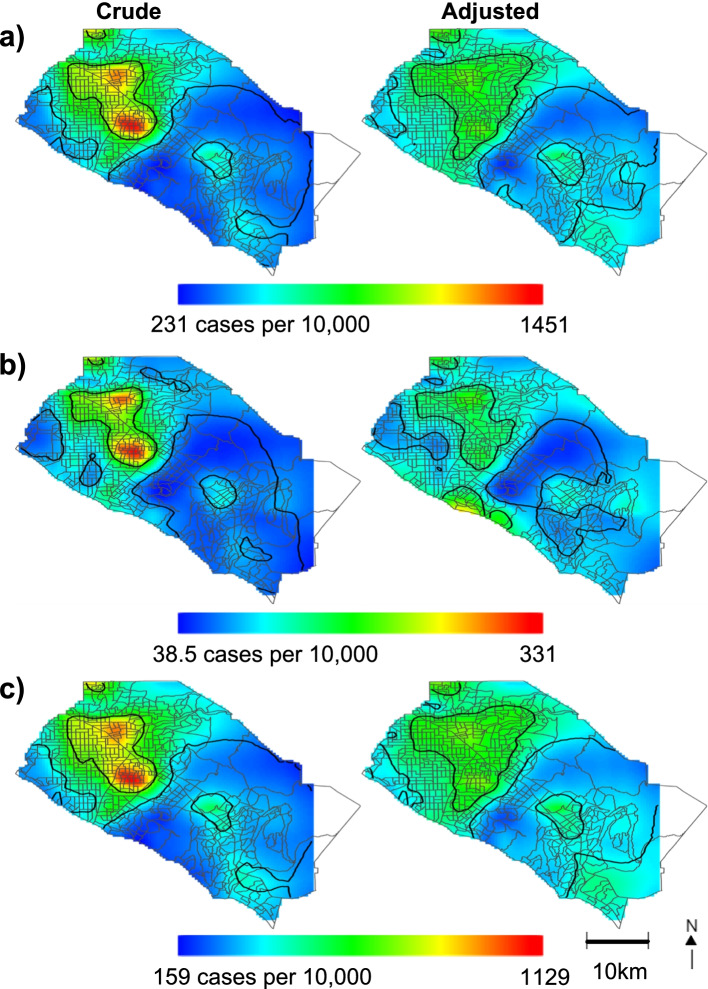
Geographic patterns of crude and adjusted incidence risk for COVID-19 cases in Orange County census tracts. **a** March 2020-Februrary 2021, **b** March 2020- August 2020, **c** September 2020-February 2021

We identified significant areas, as indicated by black contour lines, of increased and decreased risk for COVID-19 case status across OC. Statistically significant risk for COVID-19 was elevated in northern OC for the entire study period, first half, and second half of the pandemic within our crude models (Fig. 2). We observed lower risk for COVID-19 in the north-eastern corner of OC, and most parts of southern OC. After adjustment for community variables, risk decreased in northern OC but remained statistically significant; pockets of slightly increased risks were observed in this area, but they were not statistically significant. Interestingly, adjusting for our covariates significantly increased the risk for south-western beach cities along the coast during the first half of the pandemic (Fig. 2b), suggesting that there may be potential spatial confounding in that area.

Our covariates were all significantly associated with COVID-19 case status in our crude GLM and adjusted GAM models (Table 2). In our univariate models, a 10% increase of percent minority (crude RR entire study (ES): 1.19 [1.18–1.19]), and percent of population in the service industry (cRR_ES_: 1.50 [1.50,1.51]) was positively associated with COVID-19, while percent of adults 65 and older (cRR_ES_: 0.74 [0.73,0.74]), and percent educated (cRR_ES_: 0.79 [0.79, 0.79]) was negatively associated with COVID-19 in a census tract. A $10,000 increase in median household income (cRR_ES_: 0.91 [0.90,0.91]), and an increase of 1 person to average household size (cRR_ES_: 1.52 [1.51,1.52]) was also significantly associated with decreased and increased risk, respectively. The magnitude of our effect estimates was stronger in the first half of the study period compared to the second period. However, after adjusting for covariates and location in the full model (Table 2), high risk (adjusted RR range 1.05–1.10) and low risk (aRR range 0.94–0.99) covariates moved towards the null but all remained statistically significant.

**Table 2:** Univariate and adjusted risk ratios for the association between community factors and COVID-19 cases

	Univariate GLM analysis without location			Adjusted GAM analysis with location		
	Entire Study Period	Surge 1^a^	Surge 2^b^	Entire Study Period	Surge 1^a^	Surge 2^b^
N cases	1519	369	1230	1519	369	1230
Percent Minority^c^	1.19 (1.18,1.19)	1.22 (1.21,1.22)	1.18 (1.18,1.18)	1.06 (1.06,1.07)	1.07 (1.06,1.09)	1.06 (1.06,1.07)
Average Household Size	1.52 (1.51,1.52)	1.63 (1.62,1.65)	1.49 (1.48,1.50)	1.06 (1.05,1.07)	1.10 (1.08,1.13)	1.05 (1.04,1.06)
Percent Service^c^	1.50 (1.50,1.51)	1.62 (1.61,1.64)	1.48 (1.47,1.48)	1.05 (1.04,1.06)	1.06 (1.04,1.09)	1.04 (1.03,1.05)
Median Household Income^d^	0.91 (0.90,0.91)	0.89 (0.89,0.89)	0.91 (0.91,0.91)	0.98 (0.98,0.99)	0.98 (0.97,0.99)	0.99 (0.98,0.99)
Percent 65 + ^c^	0.74 (0.73,0.74)	0.66 (0.65,0.67)	0.75 (0.75,0.76)	0.97 (0.96,0.97)	0.94 (0.92,0.96)	0.97 (0.96,0.98)
Percent Educated^c^	0.79 (0.79,0.79)	0.75 (0.75,0.75)	0.80 (0.79,0.80)	-	-	-

Results are reported as risk ratios (95% confidence intervals) by census tracts, Orange County, California, March 2020- February 2021

^a^March 1, 2020- August 31, 2020

^b^September 1, 2020-February 28, 2021

^c^per 10% increase

^d^per $10,000 increase

**Fig. 3:**
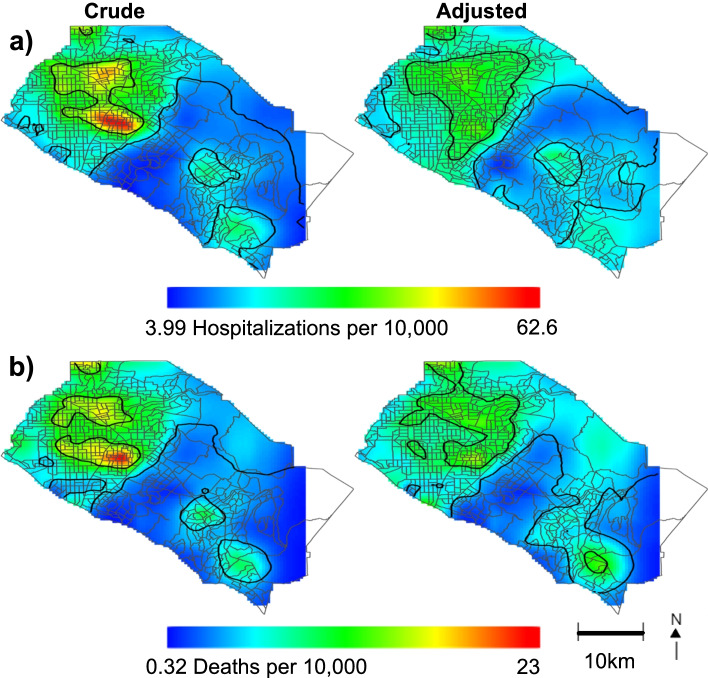
Geographic patterns of crude and adjusted incidence risk for COVID-19 **a** hospitalizations, and **b** deaths in Orange County census tracts. March 2020-Februrary 2021

Spatial risk for hospitalizations and deaths by census tract were similar to cases; risk was increased in northern OC and decreased in southern OC for crude and adjusted models (Fig. 3). We also observed a statistically significant area of increased risk in southern OC for COVID-19 deaths after adjustment (Fig. 3b). Hospitalization and death univariate GLM analyses were similar to COVID-19 cases. However, in the adjusted GAM model, percent 65 and older reversed direction and became significantly associated with elevated risk of hospitalizations (aRR_ES_: 1.17 [1.13,1.21]) and death (aRR_ES_: 1.31 [1.25,1.38]) (Table 3). Percent in service industry no longer became significant after full adjustment for hospitalizations and deaths, and median household income also became null.

**Table 3:** Univariate and adjusted risk ratios between community factors and COVID-19 hospitalizations and deaths

	Univariate GLM analysis without location		Adjusted GAM analysis with location	
	Hospitalization	Deaths	Hospitalization	Deaths
N	68	26	68	26
Percent Minority^a^	1.22 (1.21,1.23)	1.25 (1.22,1.27)	1.10 (1.08,1.13)	1.08 (1.03,1.12)
Average Household Size	1.64 (1.60,1.68)	1.65 (1.59,1.73)	1.19 (1.13,1.25)	1.19 (1.09,1.30)
Percent Service^a^	1.60 (1.56,1.64)	1.68 (1.61,1.76)	0.99 (0.94,1.03)	1.05 (0.96,1.14)
Median Household Income^b^	0.89 (0.88,0.89)	0.87 (0.86,0.88)	0.97 (0.95,0.98)	0.97 (0.95,1.00)
Percent 65 + ^a^	0.84 (0.82,0.87)	0.96 (0.92,1.01)	1.17 (1.13,1.21)	1.31 (1.25,1.38)

Results are reported as risk ratios (95% confidence intervals) by census tracts, Orange County, California, March 2020- February 2021

^a^per 10% increase

^b^per $10,000 increase

## Discussion

We observed significantly increased COVID-19 risk within census tracts in northern OC while considering community variables that may increase COVID-19 risk. Location was statistically significant even after adjustment of community variables. However, our adjusted GAM models illustrate a reduction of overall spatial risk across all three models, specifically in high risk areas in north OC. This area of high risk spatially overlays with lower-income communities and communities of color in this study. Despite a reduction in spatial patterns across our maps, residual risk and areas of statistical significance still remain after adjustment suggesting the presence of unmeasured spatial confounders. Two regions in south OC remain at moderate risk among the surrounding statistically low risk area, and areas along the center coastline became statistically significant after adjustment. This may be the result from personal perspectives from individuals who are resistant to public health interventions.

Our results also support many studies that observe health inequities with COVID-19 among disadvantaged communities [21, 32]. Within OC, census tracts with higher percentages of minority residents and service workers were associated with higher risk of COVID-19. These results are similar to a spatial study across the US which observed a greater proportion of Black individuals associated with COVID-19 and occupation was a strong predictor of cases [33]. Higher prevalence of COVID-19 were reported among Hispanic residents in Los Angeles and Orange County, potentially due to overrepresentation in industries that were considered essential during the pandemic [5, 12, 34]. This area of high risk observed in our study consists of minority communities who may also reside in crowded neighborhoods, may not be able to physically distance, and have other comorbidities that may worsen their COVID-19 prognosis [35]. An occupational study on seroprevalence among firefighters also identified a higher proportion of Hispanic firefighters who were reluctant to participate in disease surveillance activities, suggesting there may be hesitancy towards public health activities [36].

Census tracts in northern OC tended to have larger average household sizes than those of south OC. In contrast, census tracts along the southern beach and south OC were associated with higher median income, lower percent in service industry, lower percent minority, smaller household sizes, and greater percent 65 and older. Our results are consistent with a prior study in OC that observed COVID-19 incidence LISA-statistic hotspots and cold spots in northern OC and southern OC respectively. This study also observed statistically significant associations with ZIP code-level median household income and household crowding (defined as > 1 person per room) and observed shifts in risk early in the pandemic [6].

This study had a few limitations that must be considered when interpreting the results. Our analysis primarily relied on PCR positive tests and therefore only captured cases that were either symptomatic, or close contacts with other positive cases. A study in the summer of 2020 observed a seven-fold greater seroprevalence than diagnosed cases in Orange County, and we therefore may underestimate the risk of COVID within our study [5]. In addition, individuals of lower socioeconomic status are more likely to underutilize testing, limiting surveillance in already vulnerable communities [37, 38]. To our knowledge, only one other study has used GAM to investigate spatio-temporal COVID-19 trends [39]. The author was able to utilize individual-level data, which is preferable to ecologic data. We also utilized census tracts as our spatial unit of analysis and therefore subject to the modifiable areal unit problem and the uncertain geographic context problem, impacting the interpretation of our results. Furthermore, we were not able to utilize individual-level data and were limited in the available community-level data that is provided by the US Census. Data collection and surveillance at our local health department was limited, with 32% of all cases missing race/ethnicity data and 7% missing longitude and latitude, and efforts should be made to create an efficient and thorough data collection for the next epidemic that may occur. Considering the limited resources in data collection during investigations and contact tracing, there must be an ongoing discussion and collaboration with health care providers and commercial laboratories to collect more complete demographic data. In addition, increased public health funding will allow modern surveillance record systems to be deployed, which is at the root of all trend analysis and reporting.

However, we were able to identify areas that are at risk for COVID-19 infection and possibly other infectious diseases with similar determinants and parse out some of the socio-demographic factors that may increase the risk of communities. In addition, our study benefited from the use of a smaller geographic scale than prior studies and utilized novel methods to account for the effect of location and social determinants.

## Conclusions

This study observed significant areas of increased COVID-19 risk in northern census tracts, and decreased risk in central and southern census tracts in Orange County. The risk was similar across the first two surges of the pandemic, and for hospitalizations and deaths. In addition, we identified social and environmental demographics that were significantly associated with COVID-19, with percent minority, average household size, and percent in service industry contributing to COVID-19 risk, and percent sixty-five and older, and percent educated reducing COVID-19 risk. The association for percent sixty-five and older and average household size reversed for hospitalizations and deaths. After adjusting for these factors in our full models, residual spatial risk reduced in magnitude, but remained statistically significant, suggesting the presence of unaccounted environmental or demographic risk factors. Further investigation on factors that may increase risk of an infectious disease in a population is needed in order to prepare for future outbreaks.

## Data Availability

The data that support the findings of this study are available from OCHCA but restrictions apply to the availability of these data and so are not publicly available. De-identified and aggregated data are however available from the last author (ES) upon reasonable request and with permission of OCHCA.
